# Travel and the emergence of infectious diseases.

**DOI:** 10.3201/eid0102.950201

**Published:** 1995

**Authors:** M E Wilson

**Affiliations:** Harvard School of Public Health, Boston, Massachusetts, USA. mewilson@warren.med.harvard.edu

## Abstract

Travel is a potent force in the emergence of disease. Migration of humans has been the pathway for disseminating infectious diseases throughout recorded history and will continue to shape the emergence, frequency, and spread of infections in geographic areas and populations. The current volume, speed, and reach of travel are unprecedented. The consequences of travel extend beyond the traveler to the population visited and the ecosystem. When they travel, humans carry their genetic makeup, immunologic sequelae of past infections, cultural preferences, customs, and behavioral patterns. Microbes, animals, and other biologic life also accompany them. Today's massive movement of humans and materials sets the stage for mixing diverse genetic pools at rates and in combinations previously unknown. Concomitant changes in the environment, climate, technology, land use, human behavior, and demographics converge to favor the emergence of infectious diseases caused by a broad range of organisms in humans, as well as in plants and animals.


### Travel and the Emergence of Infectious Diseases

**Mary E. Wilson, M.D.**
Harvard School of Public Health and Harvard Medical School, Boston, Massachusetts, USA
Member: Harvard Working Group on New and Resurgent Infectious Diseases

*Travel is a potent force in the emergence of disease. Migration of humans has been the pathway for disseminating infectious diseases throughout recorded history and will continue to shape the emergence, frequency, and spread of infections in geographic areas and populations. The current volume, speed, and reach of travel are unprecedented. The consequences of travel extend beyond the traveler to the population visited and the ecosystem. When they travel, humans carry their genetic makeup, immunologic sequelae of past infections, cultural preferences, customs, and behavioral patterns. Microbes, animals, and other biologic life also accompany them. Today's massive movement of humans and materials sets the stage for mixing diverse genetic pools at rates and in combinations previously unknown. Concomitant changes in the environment, climate, technology, land use, human behavior, and demographics converge to favor the emergence of infectious diseases caused by a broad range of organisms in humans, as well as in plants and animals.*

Many factors contribute to the emergence of infectious diseases. Those frequently identified include microbial adaptation and change, human demographics and behavior, environmental changes, technology and economic development, breakdown in public health measures and surveillance, and international travel and commerce (1-4). This paper will examine the pivotal role of global travel and movement of biologic life in the emergence of infectious diseases. It will also examine the ways in which travel and movement are inextricably tied at multiple levels to other processes that influence the emergence of disease.

Travel is a potent force in disease emergence and spread (5). The current volume, speed, and reach of travel are unprecedented. The consequences of migration extend beyond the traveler to the population visited and the ecosystem (6). Travel and trade set the stage for mixing diverse genetic pools at rates and in combinations previously unknown. Massive movement and other concomitant changes in social, political, climatic, environmental, and technologic factors converge to favor the emergence of infectious diseases.

Disease emergence is complex. Often several events must occur simultaneously or sequentially for a disease to emerge or reemerge (Table 1) (6). Travel allows a potentially pathogenic microbe to be introduced into a new geographic area; however, to be established and cause disease a microbe must survive, proliferate, and find a way to enter a susceptible host. Any analysis of emergence must look at a dynamic process, a sequence of events, a milieu, or ecosystem.

Movement, changing patterns of resistance and vulnerability, and the emergence of infectious diseases also affect plants, animals, and insect vectors.

Address for correspondence: Mary E. Wilson, Division of Infectious Diseases, Mt. Auburn Hospital, 330 Mt. Auburn Street, Cambridge, MA 02238 USA, fax 617-965-6632; e-mail mewilson@warren.med.harvard.edu.

**Table 1. Basic concepts in disease emergence***

| |
|---|
| Emergence of infectious diseases is complex. |
| Infectious diseases are dynamic. |
| Most new infections are not caused by genuinely new pathogens. |
| Agents involved in new and reemergent infections cross taxonomic lines to include viruses, bacteria, fungi, protozoa, and helminths. |
| The concept of the microbe as *the* cause of disease is inadequate and incomplete. |
| Human activities are the most potent factors driving disease emergence. |
| Social, economic, political, climatic, technologic, and environmental factors shape disease patterns and influence emergence. |
| Understanding and responding to disease emergence require a global perspective, conceptually and geographically. |
| The current global situation favors disease emergence. |

*Adapted from Wilson ME (6).

Analysis of these species can hold important lessons about the dynamics of human disease.

To assess the impact of travel on disease emergence, it is necessary to consider the receptivity of a geographic area and its population to microbial introduction. Most introductions do not lead to disease. Organisms that survive primarily or entirely in the human host and are spread through sexual contact, droplet nuclei, and close physical contact can be readily carried to any part of the world. For example, AIDS, tuberculosis, measles, pertussis, diphtheria, and hepatitis B are easily carried by travelers and can spread in a new geographic area; however, populations protected by vaccines resist introduction. Organisms that have animal hosts, environmental limitations, arthropod vectors, or complicated life cycles become successively more difficult to "transplant" to another geographic area or population. Epidemics of dengue fever and yellow fever cannot appear in a geographic area unless competent mosquito vectors are present. Schistosomiasis cannot spread in an environment unless a suitable snail intermediate host exists in that region. Organisms that survive only under carefully tuned local conditions are less likely to be successfully introduced. Even if an introduced parasite persists in a new geographic area, it does not necessarily cause human disease. In the United States, humans infected with *Taenia solium*, the parasite that causes cysticercosis, infrequently transmit the infection because sanitary disposal of feces, the source of the eggs, is generally available. In short, the likelihood of transmission involves many biological, social, and environmental variables.

### Historical Perspective

Human migration has been the main source of epidemics throughout recorded history. William McNeill (7), in his book *Plagues and Peoples*, describes the central role of infectious disease in the history of the world. Patterns of disease circulation have influenced the outcome of wars and have shaped the location, nature, and development of human societies.

Trade caravans, religious pilgrimages, and military maneuvers facilitated the spread of many diseases, including plague and smallpox. A map in Donald Hopkins' book, *Princes and Peasants: Smallpox in History* (8), traces the presumed spread of smallpox from Egypt or India, where it was first thought to have become adapted to humans sometime before 1000 B.C. Smallpox spread easily from person to person through close contact with respiratory discharges and, less commonly, through contact with skin lesions, linens, clothing, and other material in direct contact with the patient. Because patients remained infectious for about 3 weeks, many opportunities for transmission were available. Even in this century, until the 1970s, smallpox continued to cause epidemics. A pilgrim returning from Mecca was the source of a large outbreak in Yugoslavia in the early 1970s that resulted in 174 Yugoslav cases and 35 deaths (9). The pilgrim apparently contracted the infection in Baghdad while visiting a religious site. Because his symptoms were mild, he was never confined to bed and was able to continue his travels and return home.

For most of history, human populations were relatively isolated. Only in recent centuries has there been extensive contact between the flora and fauna of the Old and New Worlds. Schoolchildren hear the rhyme "Columbus sailed the ocean blue, in fourteen hundred ninety-two," but may learn little about the disaster brought upon the native populations of the Americas by the arriving explorers. By the end of the fifteenth century, measles, influenza, mumps, smallpox, tuberculosis, and other infections had become common in Europe. Explorers from the crowded urban centers of Europe brought infectious diseases to the New World (10), where isolated populations had evolved from a relatively small gene pool and had no previous experience with many infections (11). The first epidemics following the arrival of Europeans were often the most severe. By 1518 or 1519, smallpox appeared in Santo Domingo, where it killed one-third to half of the local population and spread to other areas of the Caribbean and the Americas (10). The population of central Mexico is estimated to have dropped by one-third in the single decade following contact with the Europeans.

Travel across the Atlantic Ocean transformed the flora and fauna of the New World as well. Some of the transported materials became important sources of food (plants), clothing, and transportation (animals). Other transfers were less welcome: Japanese beetles, Dutch elm disease, and chestnut tree fungus. A.W. Crosby, exploring these exchanges between the Old and the New Worlds, sounds a pessimistic note: "The Columbian exchange has left us with not a richer but a more impoverished genetic pool" (10).

The explorers also paid a price in loss of lives from disease. Philip Curtin (12) provides a quantitative study of "relocation costs," the excess illness and death among European soldiers in the nineteenth century when they lived or worked in the tropics. Until the most recent armed conflicts, infectious diseases claimed more lives than injuries during wars.

Plague holds a prominent place in history and remains with us today. A bacterial infection caused by *Yersinia pestis*, it is primarily an infection of rodents, spread by their fleas. Human infection is incidental to the maintenance of *Y. pestis* in animal reservoirs. Yet plague periodically has erupted in human populations, wreaking great devastation, killing millions and causing infection that can be spread directly from person to person by the respiratory route. Human population movement has been essential in the spread of plague and the dispersal of rodents and their fleas to new areas. For centuries plague spread along trade routes. It reached California by boat around the turn of this century, caused epidemic infection in San Francisco, and then spread to wildlife, where it persists today in a large enzootic focus.

### Movement of People

Travel for business and pleasure constitutes a small fraction of total human movement (5,13). People migrating individually or in groups, may be immigrants, refugees, missionaries, merchant marines, students, temporary workers, pilgrims, or Peace Corps workers. Travel may involve short distances or the crossing of international borders. Its volume, however, is huge. In the early 1990s more than 500 million persons annually crossed international borders on commercial airplane flights (World Tourism Organization, Madrid, unpublished data). An estimated 70 million persons, mostly from developing countries, work either legally or illegally in other countries (14). Movement may be temporary or seasonal, as with nomadic populations and migrant workers who follow the crops. Military maneuvers worldwide employ and move huge populations. The consequences of armed conflict and political unrest displace millions. In the early 1990s, there were an estimated 20 million refugees and 30 million displaced persons worldwide (International Organization for Migration, personal communication).

Grubler and Nakicenovic estimated and plotted the average kilometers traveled daily for the French population over a 200-year period (1800-2000) and found that spatial mobility has increased more than 1000-fold (15). In the last 40 years, the size of Australia's population has doubled and the number of persons moving into and out of Australia has increased nearly 100-fold (16).

Although social, economic, and political factors push people from an area or draw them to another, environmental resources and their impact on food and water supplies are behind many conflicts leading to displacement of populations. Acute disasters, such as flooding, earthquakes, and hurricanes often force populations to seek shelter and sustenance in new lands. Chronic changes, such as drought, depletion of soil, and disappearance of fish from streams, lakes, and oceans, draw people to new territories, or, more frequently, to the fringes of large urban centers.

Another type of travel relevant to disease emergence is the shift of populations to urban areas. It is estimated that by the year 2010, 50% of the world's population will be living in urban areas. It is projected that by the year 2000, the world will comprise 24 "megacities"—sprawling metropolitan areas with populations exceeding 10 million (World Bank, UNDP, World Health Organization, unpublished data). These areas will have the population density to support persistence of some infections and contribute to the emergence of others. Many of these areas are located in tropical or subtropical regions, where the environment can support a diverse array of pathogens and vectors. Also developing are huge periurban slums, populated with persons from many geographic origins. Poor sanitation allows breeding of arthropod vectors, rodents, and other disease-carrying animals. Crowded conditions favor the spread of diseases that pass from person to person, including sexually transmitted infections. Travel between periurban slum areas and rural areas is common, paving the route for the transfer of microbes and disease. Transfer of resistance genes and genetic recombination may also occur in and spread from crowded environments of transients.

Acute disturbances, whether climatic or political, lead to interim living arrangements, such as refugee camps and temporary shelters, that provide ideal conditions for the emergence and spread of infections. Temporary living quarters often share similarities with periurban slums: crowding, inadequate sanitation, limited access to medical care, lack of clean water and food, dislocation, multiethnic composition, and inadequate barriers from vectors and animals. An example is the movement of 500,000-800,000 Rwandan refugees into Zaire in 1994. Almost 50,000 refugees died during the first month as epidemics of cholera and *Shigella dysenteriae* type 1 swept through the refugee camps (17).

Movement into a rural environment poses different risks and often places new rural populations in contact with pathogens that are in the soil and water or are carried by animals or arthropods (18). Some of these pathogens such as Guanarito (19) and Sabià viruses (20) in South America, were only recently recognized as capable of infecting humans.

### Consequences of Movement

Human migration favors the emergence of infectious diseases through many mechanisms. When people migrate, they carry their genetic makeup, their accumulated immunologic experience, and much more (Table 2). They may carry pathogens in or on their bodies and may also transport disease vectors, such as lice. Their technology (agricultural and industrial), methods for treating disease, cultural traditions, and behavioral patterns may influence their risk for infection in a new environment and their capacity to introduce disease into the new region. Their social standing and resources may affect their exposure to local infections and their access to adequate nutrition and treatment. People also change the environment in many ways when they travel or migrate—they plant, clear land, build, and consume. Travel is relevant in the emergence of disease if it changes an ecosystem. The following examples show the many ways in which migration can influence the emergence of disease in a new area.

1. Humans may carry a pathogen in a form that can be transmitted, then or later, directly or indirectly to another person. The pathogen may be silent (during the incubation period, chronic carriage, or latent infection) or clinically evident. Examples include hepatitis B virus, human immunodeficiency virus (HIV), *Mycobacterium tuberculosis*, *M. leprae*, *Salmonella typhi*, and other salmonella. Disease may be especially severe when a pathogen is introduced into a population that has no previous exposure to the infection. How long the consequences of migration persist varies with the specific infection. The two most critical characteristics are the duration of survival of the pathogen in a potentially infective form and its means of transmission.

2. Epidemic cholera in Africa spread along the West African coast and, when the disease moved inland, followed fishing and trading routes. Markets, funerals, refugee camps—events that involved migration of persons and large gatherings with close contact—helped spread the infection. With El Tor cholera, asymptomatic and mild infections can outnumber severe disease by 100 to 1 (21), thus permitting those infected to continue to move and work.

3. Pilgrims carried an epidemic strain of group A *Neisseria meningitidis* from southern Asia to Mecca in 1987. Other pilgrims who became colonized with the epidemic strain introduced it into sub-Saharan Africa, where it caused a wave of epidemics in 1988 and 1989 (22).

4. Humans may carry a pathogen that can be transmitted only if conditions are permissive. This permissiveness can pertain to human behavior, the environment, or the presence of appropriate vectors or intermediate hosts. For example, the ease with which HIV spreads in a population depends on sexual practices, condom use, the number of sex partners, and intravenous drug use, among other factors. Malaria requires specific mosquito vectors (with access to susceptible humans) to spread to new geographic regions. Schistosomiasis can be introduced into a new region only if the appropriate snail host is present and if the eggs excreted (in urine or feces, from an infected person), reach the snails in an appropriate environment.

**Table 2. What is carried by humans into new regions?**

| |
|---|
| Pathogens in or on body |
| Microbiologic flora |
| Vectors on body |
| Immunologic sequelae of past infections |
| Vulnerability to infections |
| Genetic makeup |
| Cultural preferences, customs, behavioral patterns, technology |
| Luggage and whatever it contains |

5. Humans may carry a strain of microbe that has an unusual resistance pattern or virulence genes. A multiple-drug-resistant strain of *Klebsiella pneumoniae* appears to have been transferred by an asymptomatic woman from a hospital in Bahrain to Oxford, where it caused outbreaks in two British hospitals (23). People also carry their background flora, in the intestinal tract, for example, which may contain plasmids and resistance genes that can interact with microbes in a new area. It is not just the classic pathogens that may be relevant to the emergence of a new disease but the individual traveler's total microbiologic "baggage."

6. Visitors to a region may lack immunity to locally endemic infections, such as hepatitis A and sand-fly fever. Visitors may suffer severe or different manifestations of infection or disease at an age when the local population is immune to it. Resettlement of populations into malaria-endemic regions can lead to a high death rate from falciparum malaria.

7. Kala-azar caused a deadly outbreak in remote villages in southern Sudan in 1994. The origin was thought to be the villagers' exposure to the sand-fly vector during migration to a food distribution center that had been established by a relief organization (24). The migration took a malnourished population from a nonendemic zone into the southern part of the kala-azar–endemic zone. Unfamiliarity with the disease and the poor nutritional status of the population probably contributed to a high death rate (24).

8. Behavioral patterns in a new region may place visitors at risk for infection, while the local population, possibly because of their knowledge of disease risks, may not be at risk. Behavior patterns may involve food preparation (such as eating some foods raw), clothing (or lack of it), (for example, going barefooted), sleeping arrangements (sleeping on the ground or out of doors in an unscreened area), and contact with animals.

9. Susceptibility of a population may vary because of genetic differences. A microbe introduced into a new region may have a greater or lesser impact, depending on the host population. Genetic factors influence susceptibility to and expression of several infectious diseases. Although these interactions are not yet well defined for most infections, genetic factors influence infections caused by different classes of organisms, including cholera (25,26), parvovirus infection (27), malaria, and *Helicobacter pylori* infection (28).

To determine the consequences of travel both the traveler and the population visited must be considered. Migration may be in only one direction, though travel often involves returning to the point of origin, perhaps after the traveler has made many stops along the way. The changes in the various ecosystems as a consequence of the migration guide the emergence of diseases; any study that simply focuses on the traveler is too narrow.

The distance traversed is less important than the differences in biological life in different areas and differences in receptivity and vulnerability. In thinking about disease emergence, what matters is the potential of a disease to appear in a place, population, or extent not previously reported.

What is the long-term impact of migration and travel on human disease? Carriage of pathogens is only part of the influence on disease emergence. Introduced technology, farming methods, treatment and drugs, chemicals, and pesticides may have a far greater and longer impact on disease patterns in a region than the life of a person. Deforestation, building of dams, and opening of roads into previously inaccessible areas have all been associated with population movements and changes in distribution and frequency of a variety of infections in humans (such as malaria, schistosomiasis, Rift Valley fever, and sexually transmitted diseases).

Increasingly the vehicle of transportation is the site or even the source of outbreaks. During travel, people from diverse origins are enclosed in close proximity for a hours or days and then discharged to move on to many distant places. These temporary new habitats, jumbo jets or huge ocean liners, can be the sites for dissemination of the microbes (as happens, for example with *Legionella pneumophila* infections (29), foodborne infections, and cholera) or provide a milieu for person-to-person transmission (influenza, tuberculosis (30,31)).

### Shipping and Commerce

The biomass of humans constitutes only a fraction of the matter moved about the earth. Humans carry and send a huge volume of plants, animals and other materials all over the face of the globe. Much of this movement results from the planned transport of goods from one place to another, but some is an unintended consequence of shipping and travel. All has an impact on the juxtaposition of various species in different ecosystems. "Hitchhikers" include all manner of biologic life, both microscopic and macroscopic. Animals can carry potential human pathogens and vectors. The globalization of markets brings fresh fruits and vegetables to dinner tables thousands of miles from where they were grown, fertilized, and picked. Tunnels, bridges, and ferries form means to traverse natural barriers to species spread. The roads built to transport people often speed the movement of diseases from one area to another. Mass processing and wide distribution networks allow for the amplification and wide dissemination of potential human microbes.

Examples of introduced species include plants and animals—insects, microbes, and marine organisms.

1. Ships convey marine organisms on their hulls and in their ballast water. For example, 367 different species were identified in ballast water of ships traveling between Japan and Coos Bay, Oregon (32). Introductions have had devastating effects in some areas, for example such as the Black and Azov seas, where newly introduced jellyfishlike creatures called ctenophores have ruined local fishing (33).

2. *Vibrio cholerae* may have been introduced to South America by shipping (34). Researchers isolated the organism in samples of ballast, bilge, and sewage from 3 of 14 cargo ships docked at Gulf of Mexico ports. The ships had last ports of call in Brazil, Colombia, and Chile (35). *V. cholerae* O1, serotype Inaba, biotype El Tor, indistinguishable from the Latin American epidemic strain, was found in oysters and oyster-eating fish from closed oyster beds in Mobile Bay, Alabama (36). *V. cholerae* O139 has spread along waterways in Asia, although the people carried on the boats doubtless played a role (37,38).

3. *Aedes albopictus* was introduced into the United States inside used tires shipped from Asia (39,40). The mosquito's introduction causes concern because it is an aggressive biter, survives in both forest and suburban habitats, and appears to be a competent vector for several human pathogens. It has been associated with epidemic dengue fever transmission in Asia and is a competent laboratory vector of La Crosse, yellow fever, and other viruses (41). In Florida, 14 strains of eastern equine encephalitis virus have been isolated from *A. albopictus* (42). The mosquito is now established in at least 21 of the contiguous states in United States and in Hawaii.

4. The African anopheles mosquitoes arrived in Brazil in about 1929. This vector could breed under conditions other New World mosquitoes could not. Although the malaria parasite was already found in Brazil, this new vector expanded the range of transmission. An estimated 20,000 persons died of malaria before the introduced anopheles mosquitoes were eliminated.

5. It has been repeatedly demonstrated that mosquitoes are present—and survive—on international flights. In random searches of airplanes in London, mosquitoes were found on 12 of 67 airplanes from tropical countries (43). Arthropods can survive even more extreme environments. In one study, mosquitoes, house flies, and beetles placed in wheel bays of Boeing 747B aircraft survived flights of 6-9 hours with external temperatures of -42° C (43). Airplanes have also carried infective mosquitoes that caused human infection outside malaria-endemic areas (in Europe, for example).

6. Vehicles can transport vectors over land. *Glossina palpalis*, a vector for African trypanosomiasis (sleeping sickness), can fly up to 21 km but can be transported much longer distances on animals and in land vehicles.

7. Seven persons in Marburg, Germany, died after handling blood and tissues from African green monkeys from Uganda. The tissues contained an organism later named Marburg virus (44).

8. Exotic animals transported from their usual habitats are clustered in zoos; others are used in research laboratories where they have occasionally caused severe disease in humans. Two examples are B virus from primates (45) and hemorrhagic fever with renal syndrome from rodents (46).

9. The world trade and globalization of organs, tissues, blood, and blood products is growing. Researchers are considering animals as sources for tissues and organs for transplantation (47).

10. Plants may not directly cause human disease. But they can alter an ecosystem and facilitate the breeding of a vector for human disease. This can also displace traditional crops that provide essential nutrition. Vertical transmission of plant pathogens (and spread of plant diseases) can result from seed movement (48). Carriage of seeds into new areas can introduce plant pathogens.

11. Migration and altered environments have increased the so-called weedy species. These species migrate easily and have high rates of reproduction. If they lack local predators, they can displace other species and often upset local ecology.

### Introduction of Species into New Areas

Introducing species into new geographic areas is not new, but the current volume and frequency of introductions are unprecedented. A pathogen's survival and spread in a new environment are determined by its basic reproductive rate, which is the average number of successful offspring a parasite can produce (49). To invade and establish itself in a host population, a parasitic species must have a basic reproductive rate exceeding one (49). The simplicity of this statement belies the complexity of circumstances that influence invasion and persistence. These circumstances encompass biological, social, and environmental factors.

As noted already, factors that can influence receptivity include climate and environmental conditions, sanitation, socioeconomic conditions (50), behavior, nutrition, and genetics. *V. cholerae* persists in an aquatic reservoir off the Gulf Coast of the United States, yet epidemic cholera has not been a problem in the United States. Where poverty and poor sanitation prevail, the presence of *V. cholerae* can be a source of endemic disease and periodic epidemics.

Disease emergence is often complex. An outbreak of malaria in San Diego, California, occurred when parasitemic migrant workers were employed in an area where mosquitoes capable of transmitting malaria had access to the workers and to a susceptible human population (51). Many conditions had to be met to allow transmission.

Migration may introduce parasites into an area where a different intermediate host or vector could change the incidence of disease. Cycling through a different host can lead to different transmission rates, different infectivity, and even different clinical expression. A parasite may be more successful in a new site because of a larger susceptible population or the absence of predators.

### Confluence of Events

Massive global travel is taking place simultaneously with many other processes that favor the emergence of disease. For example, the human population is more vulnerable because of aging, immunosuppression from medical treatment and disease (such as AIDS), the presence of prostheses (e.g., artificial heart valves and joints), exposure to chemicals and environmental pollutants that may act synergistically with microbes to increase the risk of diseases, increased poverty, crowding and stress, and increased exposure to UV radiation. Technologic changes, while providing many benefits, can also promote disease dissemination. Resistance of microbes and insects to antimicrobial drugs and pesticides interferes with the control of infections and allows transmission to continue. Changes in land use can alter the presence and abundance of vectors and intermediate hosts.

Microbes are enormously resilient and adaptable. They have short life spans, which allow rapid genetic change. Humans, by comparison, are slow to change genetically but can change their behavior. People move and construct barriers to prevent contact with microparasites, macroparasites, and the extremes of the environment. Technology fosters a perception of human invincibility but actually creates new vulnerabilities, as it enables us to go deeper, higher, and into more remote and hostile environments. Studies show that no place on earth is devoid of microbes. Their range and resiliency are truly phenomenal. Only a fraction of the existing microbes have been characterized. Travel and exploration provide a greater opportunity for humans to come into unsampled regions with these uncharacterized microbes.

### Summary and Conclusions

Global travel and the evolution of microbes will continue. New infections will continue to emerge, and known infections will change in distribution, severity and frequency. Travel will continue to be a potent factor in disease emergence. The current world circumstances juxtapose people, parasites, plants, animals, and chemicals in a way that precludes timely adaptation. The combination of movement at many levels and profound change in the physical environment can lead to unanticipated diseases spread by multiple channels. In many instances, the use of containment or quarantine is not feasible. Research and surveillance can map the global movement and evolution of microbes and guide interventions. Integration of knowledge and skills from many disciplines—the social, biological, and physical sciences—is needed. The focus should be system analysis and the ecosystem rather than a disease, microbe, or host.

*Dr. Wilson is Chief of Infectious Diseases at Mount Auburn Hospital in Cambridge and Assistant Professor of Population and International Health and Epidemiology at the Harvard School of Public Health. An active participant in the Harvard Working Group on New and Reemergent Infectious Diseases since its inception in 1991, she is the senior editor, with Richard Levins and Andrew Spielman, of* Disease in Evolution: Global Changes and Emergence of Infectious Diseases *(3), a book based on the 1993 Woods Hole workshop on emerging infections.*

### References

1. Lederberg J, Shope RE, Oaks SC, Jr., eds. Emerging infections: microbial threats to health in the United States. Washington, D.C.: National Academy Press, 1992.
2. Centers for Disease Control and Prevention. Addressing emerging infectious disease threats: a prevention strategy for the United States. Atlanta: U.S. Department of Health and Human Services, 1994.
3. Wilson ME, Levins R, Spielman A. Disease in evolution: global changes and emergence of infectious diseases. New York: New York Academy of Sciences, 1994;740.
4. Levins R, Awerbuch T, Brinkmann U, et al. The emergence of new diseases. American Scientist 1994;82:52-60.
5. Wilson ME. A world guide to infections: diseases, distribution, diagnosis. New York: Oxford University Press, 1991.
6. Wilson ME. Disease in evolution: introduction. In: Wilson ME, Levins R, Spielman A, eds. Disease in evolution: global changes and emergence of infectious diseases. New York: New York Academy of Sciences, 1994;740:1-12.
7. McNeill WH. Plagues and peoples. Garden City, N.Y.: Anchor Press/Doubleday, 1976.
8. Hopkins DR. Princes and peasants: smallpox in history. Chicago: University of Chicago Press, 1983.
9. World Health Organization. Smallpox: Yugoslavia. Wkly Epidemiol Rec 1972;47:161-2.
10. Crosby AW, Jr. The Columbian exchange. Westport, Conn. Greenwood Press, 1972:219.
11. Black FL. Why did they die? Science 1992;258:1739-40.
12. Curtin PD. Death by migration: Europe's encounter with the tropical world in the nineteenth century. Cambridge, U.K.: Cambridge University Press, 1989.
13. Bradley DJ. The scope of travel medicine: an introduction to the conference on international travel medicine. In: Steffen R, Lobel HO, Haworth J, Bradley, eds. Travel Medicine. Berlin: Springer-Verlag, 1989:1-9.
14. Siem H, Bollini P, eds. Migration and health in the 1990s. International Migration 1992;30.
15. Grubler A, Nakicenovic N. Evolution of transport systems. Laxenburg, Vienna: ILASA, 1991.
16. Haggett P. Geographical aspects of the emergence of infectious diseases. Geogr Ann 1994;76 B(2):91-104.
17. Goma Epidemiology Group. Public health impact of Rwandan refugee crisis: what happened in Goma, Zaire, in July, 1994? Lancet 1995;345:339-44.
18. Meslin F.-X. Surveillance and control of emerging zoonoses. World Health Stat Q 1992;45:200-7.
19. Tesh RB, Jahrling R, Salas R, Shope RE. Description of Guanarito virus (Arenaviridae: *Arenavirus*), the etiologic agent of Venezuelan hemorrhagic fever. Am J Trop Med Hyg 1994;50:452-9.
20. Coimbra TLM, Nassar ES, Burattini NM, et al. A new arenavirus isolated from a fatal case of haemorrhagic fever in Brazil. Lancet 1994;343:391-2.
21. Glass RI, Claeson M, Blake PA, Waldman RJ, Pierce NR. Cholera in Africa: lessons on transmission and control for Latin America. Lancet 1991;338:791-5.
22. Moore PS, Reeves MW, Schwartz B, Gellin BG, Broome CV. Intercontinental spread of an epidemic group A *Neisseria meningitidis* strain. Lancet 1989;2:260-3.
23. Cookson B, Johnson AP, Azadian B, et al. International inter- and intrahospital patient spread of a multiple antibiotic-resistant *Klebsiella pneumoniae.* J Infect Dis 1995;171:511-3.
24. Mercer A, Seaman J, Sondorp E. Kala azar in eastern Upper Nile Province, southern Sudan. Lancet 1995;345:187-8.
25. Glass RI, Holmgren I, Haley CE, et al. Predisposition to cholera of individuals with O blood group. Am J Epidemiol 1985;121:791-6.
26. Clemens JD, Sack DA, Harris JR, et al. ABO blood groups and cholera: new observations on specificity of risk and modifications of vaccine efficacy. J Infect Dis 1989;159:770-3.

27. Brown KE, Hibbs JR, Gallinella G, et al. Resistance to parvovirus B19 infection due to lack of virus receptor (erythrocyte P antigen). N Engl J Med 1994:330:1192-6.
28. Boren T, Falk P, Roth KA, Larson G, Normark S. Attachment of *Helicobacter pylori* to human gastric epithelium mediated by blood group antigens. Science 1993;292:1982-95.
29. Centers for Disease Control and Prevention. Update: outbreak of Legionnaires' disease associated with a cruise ship. MMWR 1994;43:574-5.
30. Driver DR, Valway SE, Morgan M, Onorato IM, Castro KG. Transmission of *Mycobacterium tuberculosis* associated with air travel. JAMA 1994;272:10311-35.
31. Centers for Disease Control and Prevention. Exposure of passengers and flight crew to *Mycobacterium tuberculosis* on commercial aircraft, 1992-1995. MMWR 1995;44:137-40.
32. Carlton JT, Geller JB. Ecological roulette: the global transport of non-indigenous marine organisms. Science 1993;261:78-82.
33. Travis J. Invader threatens Black, Azov Seas. Science 1993;262:1366-7.
34. World Health Organization. Cholera in the Americas. Wkly Epidemiol Rec. 1992;67:33-9.
35. McCarthy SA, McPhearson RM, Guarino AM. Toxigenic *Vibrio cholerae* O1 and cargo ships entering Gulf of Mexico. Lancet 1992;339:624-5.
36. DePaola A, Capers GM, Moters ML, et al. Isolation of Latin American epidemic strain of *Vibrio cholerae* O1 from US Gulf Coast. Lancet 1992;339:624.
37. Ramamurthy T, Garg S, Sharma R, et al. Emergence of novel strain of *Vibrio cholerae* with epidemic potential in southern and eastern India. Lancet 1993;341:703-4.
38. Albert MJ, Siddique AK, Islam MS, et al. Large outbreak of clinical cholera due to *Vibrio cholerae* non-O1 in Bangladesh. Lancet 1993;341:704.
39. Reiter P, Sprenger D. The used tire trade: a mechanism for the worldwide dispersal of container-breeding mosquitoes. J Am Mosq Control Assoc 1987;3:494-501.
40. Craven RB, Eliason DA, Francy P, et al. Importation of *Aedes albopictus* and other exotic mosquito species into the United States in used tires from Asia. J Am Mosq Control Assoc 1988;4:138-42.
41. Moore CG, Francy DB, Eliason DA, Monath TP. *Aedes albopictus* in the United States: rapid spread of a potential disease vector. J Am Mosq Control Assoc 1988;4:356-61.
42. Mitchell CJ, Niebylski ML, Smith GC, et al. Isolation of eastern equine encephalitis virus from *Aedes albopictus* in Florida. Science 1992;257:526-7.
43. Russell RC. Survival of insects in the wheel bays of a Boeing 747B aircraft on flights between tropical and temperate airports. Bull WHO 1987;65:659-62.
44. Martini GA, Siegert R, eds. Marburg virus disease. Berlin:Springer-Verlag, 1971.
45. Weigler BJ, Hird DW, Hilliard JK, Lerche NW, Roberts JA, Scott LM. Epidemiology of cercopithecine herpesvirus 1 (B virus) infection and shedding in a large breeding cohort of rhesus macaques. J Infect Dis 1993;167:257-63.
46. Desmyter J, LeDuc JW, Johnson KM, Brasseur F, Deckers C, van Ypersele de Strihou C. Laboratory rat-associated outbreak of haemorrhagic fever with renal syndrome due to Hantaan-like virus in Belgium. Lancet 1983;ii:1445-8.
47. Fishman JA. Miniature swine as organ donors for man: strategies for prevention of xenotransplant-associated infections. Xenotransplantation 1994;1:47-57.
48. Anderson PK, Morales FJ. The emergence of new plant diseases: the case of insect-transmitted plant viruses. In: Wilson ME, Levins R, Spielman A, eds. Disease in evolution: global changes and emergence of infectious diseases. New York: New York Academy of Sciences, 1994;740:181-94.
49. Anderson RM, May RM. Infectious diseases of humans: dynamics and control. Oxford, U.K.: Oxford University Press, 1991.
50. Spence DPS, Hotchkiss J, Williams CSD, Davies PDO. Tuberculosis and poverty. Br Med J 1993;307:759-61.
51. Maldonado YA, Nahlen BL, Roberto RR, et al. Transmission of *Plasmodium vivax* malaria in San Diego County, California, 1986. Am J Trop Med Hyg 1990;42:3-9.
